# Fluorescence Naphthalene Cationic Schiff Base Reusable Paper as a Sensitive and Selective for Heavy Metals Cations Sensor: RSM, Optimization, and DFT Modelling

**DOI:** 10.1007/s10895-023-03426-6

**Published:** 2023-09-15

**Authors:** Salah M. Tawfik, Ahmed A. Farag, Ali A. Abd-Elaal

**Affiliations:** Egyptian Petroleum Research Institute, Cairo, 11727 Egypt

**Keywords:** Naphthalene cationic Schiff base, Paper-based sensor, Color shift, Mn^2+^and Co^2+^ sensor, RSM, DFT

## Abstract

Heavy metals are particularly damaging contaminants in the environment, and even trace concentrations represent a risk to human health due to their toxicity. To detect the heavy metals of Mn^2+^ and Co^2+^ ions, a novel selective reusable paper-based Fluorescence naked-eye sensor based on naphthalene cationic Schiff base (NCSB) was synthesized and confirmed using FT-IR, ^1^ H-NMR, and MS tools. Based on a blue to colorless color change in the aqueous solution, the NCSB sensor is utilized to Mn^2+^ and Co^2+^ cations selectively among other metal ions (Fe^2+^, Cu^2+^, Mg^2+^, Ni^2+^, Zn^2+^, Cd^2+^, Hg^2+^, Pb^2+^, Sn^2+^ and Cr^3+^). In the aqueous medium, the NCSB sensor displayed high sensitivity, with limits of detection (LOD) values of 0.014 µM (14.08 nM) and 0.041 µM (41.47 nM) for Mn^2+^ and Co^2+^ cations, respectively. The paper-based sensor naked-eye detected Mn^2+^ and Co^2+^ cations in water at concentrations as low as 0.65 µM (65 nM) and 0.086 µM (86 nM), respectively. It was discovered that 5 min of incubation time and a pH range of 7 to 11 were optimal for the complexation reaction between the Mn^2+^ and Co^2+^ ions and the NCSB sensor. Through a static quenching process, the interaction of the different metal ions with the Schiff base group in the NCSB molecule results in the development of a ground-state non-fluorescent complex. NCSB sensor was also successfully applied in analysis of Mn^2+^ and Co^2+^ in environmental water with good recoveries of 94.8–105.9%. The theoretical calculations based on density functional theory (DFT) studies are in support of experimental interpretations. The links between the input factors and the anticipated response were evaluated using the quadratic model of the response surface methodology (RSM) modeling.

**Supplementary Information:**

The online version contains supplementary material available at 10.1007/s10895-023-03426-6.

## Introduction

**Scheme 1:**
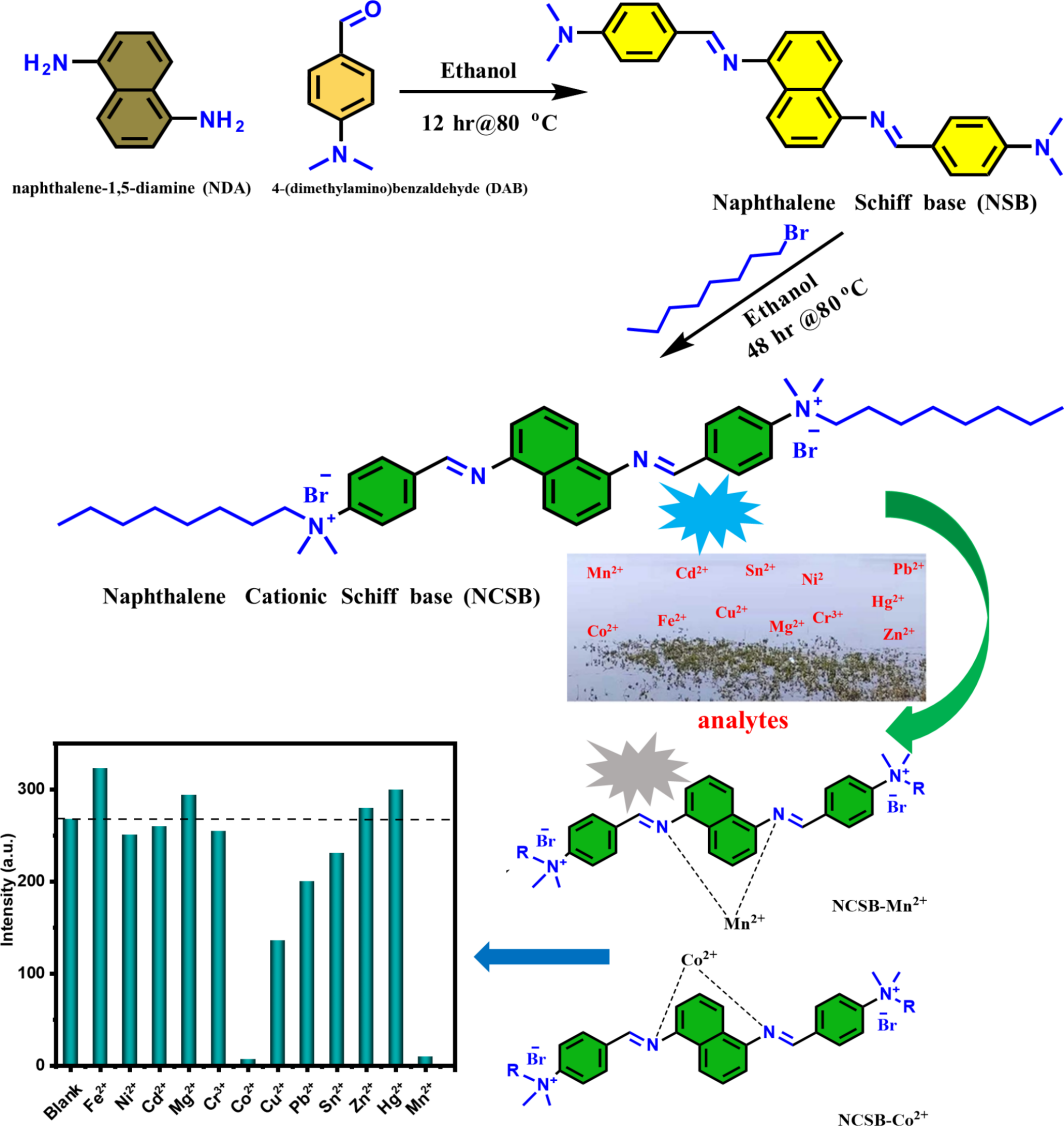
Schematic Representation of Synthesis and Mn^2+^, Co^2+^ Ions Detection Properties of NCSB.

Heavy metals, which pose a serious risk to human health and the environment, access the ecosystem through a variety of industries, including lead batteries, paint, dyes, mining, and industrial facilities [1–4]. At low concentrations, some heavy metals (e.g., nickel, cobalt, and copper), are necessary nutrients for life processes; however, large concentrations of these cations can be harmful to living cells [5–7]. Cobalt (Co^2+^) excessive accumulation can result in abnormal thyroid function, caused genotoxicity and cell death, red blood cell disparity, rising carcinogenic impacts, and lung diseases, all of which can result in mortality in humans [8]. Furthermore, manganese ion (Mn^2+^) is an essential element for the human body and plays important roles in metabolism and other biological processes in normal concentrations [9]. On the contrary overexposure to Mn^2+^ is harmful, particularly to the nervous system, causing adverse central nervous system disorders such as Parkinson’s disease [10]. Furthermore, Mn^2+^ ions deficiency can cause delayed blood coagulation and hypercholesterolemia [9]. Consequently, the new simple, and sensitive method for determining Mn^2+^ is essential [11]. As a result, detecting Mn^2+^ and Co^2+^ ions in environmental matrices are critical to life quality as well as the advancement of highly specific and sensitive chemosensor for these metals contribute significantly to one of the United Nations’ objectives for sustainable development. Recently fluorescence-based detection received a lot of attention due to a good color change because of interaction with different targets, in addition to visual naked-eye detections [6, 12–14]. Metals have been determined using conventional methods such as atomic absorption spectroscopy [15]. cyclic voltammetry [16], inductively coupled plasma spectrometry [17]. Despite these methods are extremely precise for identifying targeted cations, they are inconvenient and difficult to use because they require expensive and complicated instruments [18]. As a result, the development of a highly specific fluorescent device is required to create an easy-to-perform, fast, and cost-effective detection platform for environmental heavy metal cations. Considering its optoelectronic properties, such as noticeable absorption and emission spectra, the naphthalene Schiff base compound was used as a fluorophore for designing fluorescent sensors [19]. Furthermore, previous studies have shown that naphthalene-containing Schiff bases can selectively detect various cations due to the imine group (C = N) having a robust binding for divalent ions [20, 21]. There have recently been papers on the optical sensing of Al^3+^, Zn^2+^, and Cu^2+^ using this system [22, 23], showing that naphthalene Schiff base molecules are extensively utilized in the development of advanced sensors that are specific to heavy metal. In this work, the use of a new Schiff base cationic surfactant-based naphthalene having R_4_N^+^ and C = N binding sites to design new cationic sensor and their applications for analyte detection illustrate the high novelty of this work [24] (Scheme 1). By conducting a competitive study (Table 1), this method has the following merits: (1) high sensitivity and selectivity to differentiate the Mn^2+^ and Co^2+^ ions from other heavy metal ions; (2) this kind of cationic surfactant characterized with hydrophobic π-conjugated backbones, hydrophobic long alkyl chain, and hydrophilic quaternary ammonium group, thereby endowing them with outstanding features such as solubility in water, strong optical properties patrimonial from their conjugated backbones, and self-assembly behavior; (3) the integration of three different moieties can provide a noteworthy fluorescence quenching based on multiple points of interactions; (4) Our developed method possesses a rapid detection time (5 min) and doesn’t require any expensive or complicated procedures; (5) Importantly, the designed sensor was integrated with paper-based device and smartphone for portable and onsite Mn^2+^ and Co^2+^ sensing in aqueous solution; (6) Density functional theory (DFT) studies-based theoretical computations validate experimental interpretations.

**Table 1:** Comparison of analytical performance of current Mn^2+^ and Co^2+^ methods

Sensor materials	Samples	Target	LOD, µM	Linear range µM	Ref.
Luminol chemiluminescence	copper chelates	Co^2+^ Mn^2+^	0.00024 0.375	----------	[60]
Microfluidic paper-based analytical devices	drinking, tap, and pond water	Co^2+^ Mn^2+^	10 2.0	21.8–174.4 4.4–22	[61]
Anionic pyridylazo dye	aqueous media	Mn^2+^	7.28	0–9	[62]
Melamine based poly(azomethine–urethane)	water	Mn^2+^	0.15	0-4 × 10^4^	[63]
Diaminomaleonitrile	Tap water	Mn^2+^	0.18	0–60	[64]
9 H–carbazole derivative	water	Mn^2+^	10	0–60	[65]
A portable sequential injection analysis device	Tap water	Mn^2+^	0.011	0.1-8	[66]
Bimetallic Ag–Au nanoparticles	Pharmaceutical	Mn^2+^	0.018	0.02–0.2	[67]
Gold Nanoparticle Modified with Azomethine	Water	Co^2+^	0.083	0.02–1.6	[68]
Schiff base ligand	Petroleum water	Mn^2+^	0.1 and 0.037	0.037 − 0.01	[69]
Multiresponsive Schiff base	river water	Co^2+^	0.001	0-0.01	[70]
Thermosensitive fluorescent microgels	tap and lake water	Co^2+^	0.247	0–0.055	[71]
Naphthalene Cationic Schiff Base	tap and river water	Mn^2+^ Co^2+^	0.014 0.041	0.006–0.125 0.0125–0.125	This wok

## Experimental Section

### Materials and Instruments

All materials and reagents used in this study were analytical grade obtained from Sigma Aldrich and used as its. The Stuart SMP30 (115/230 VAC, integrated cooling 350 °C to 50 °C in 10 min, ‎United Kingdom) melting point instrument was used to determine the melting points of **NSB** and **NCSB**. FTIR spectra of **NSB** and **NCSB** were obtained using an FT/IR-6300 Fourier Transform Infrared Spectrometer (Jasco, Japan). A Bruker NMR instrument was used to record ^1^ H NMR spectra at 400 MHz of **NSB** and **NCSB**. The **NCSB** sensor’s mass spectra were recorded using an LC-MS Spectrometer Model Q-ToF Micro Waters. The fluorescence experiments were carried out on a Jasco FP-6500 spectrometer with a quartz cuvette recorded at 400 MHz using a Bruker NMR instrument. The Mass spectra of the surfactant were recorded on MS Spectrometer Model Q-ToF Micro Waters. The fluorescence experiments were performed on an FP-6500 spectrofluorometer (Jasco, Japan) using a quartz cuvette with a 1-cm path length. The absorption spectra were obtained using an Agilent 8543 (Agilent, USA) UV/Vis spectrophotometer.

### Fluorescence Sensor Synthesis

**Fig. 1:**
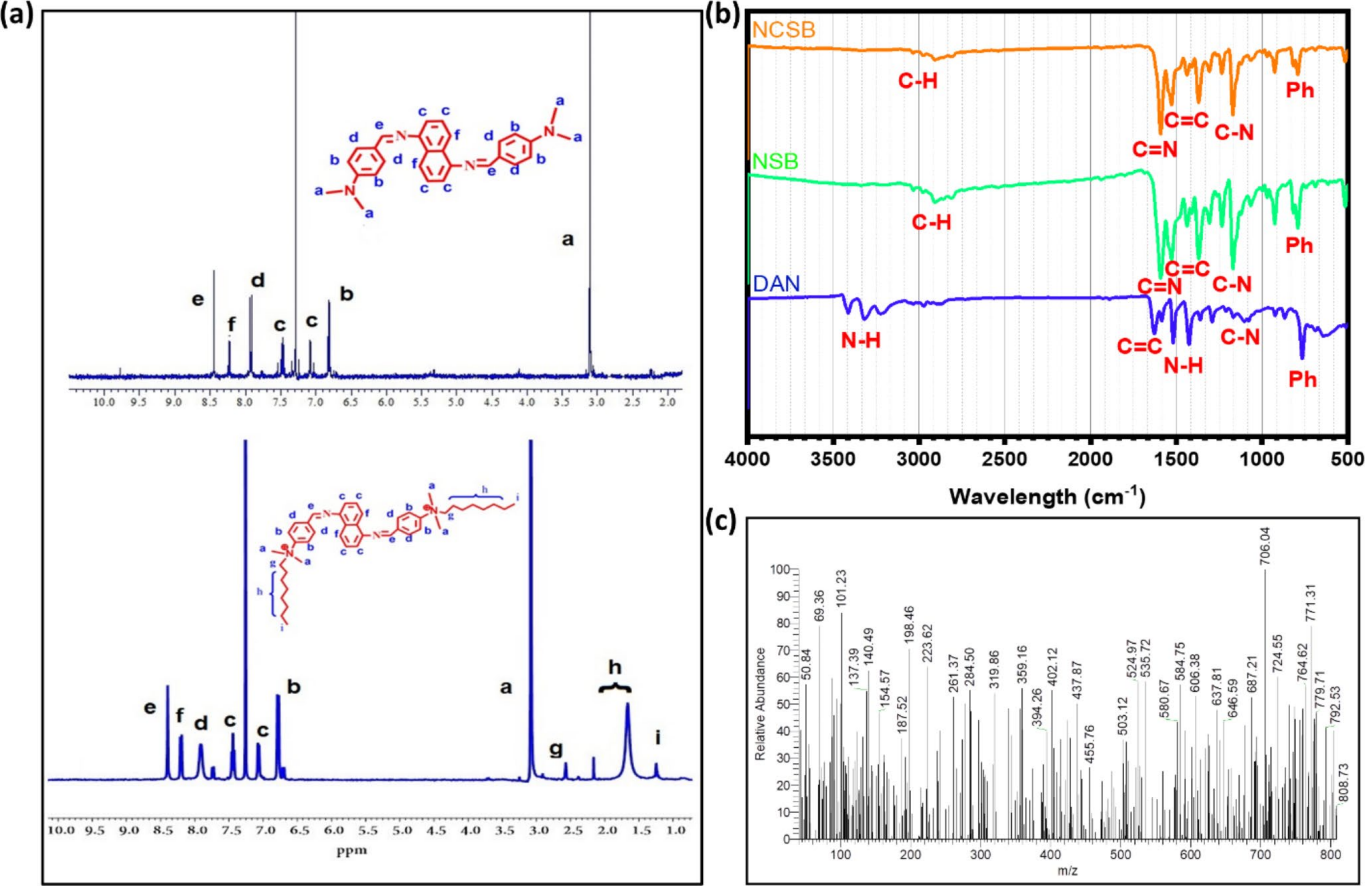
Characterization charts of: **(a)**^1^ H-NMR spectra of NSB and NCSB, **(b)** FTIR comparison of DAN, NSB and NCSB, **(c)** MS (m/z) spectra of NCSB.

Scheme 1 represented the synthetic pathway for the synthesis of NCSB surfactant. Figure 1 depicts the FTIR and ^1^ H-NMR charts used to validate the synthesized sensor.

#### Synthesis of 4,4’-((1Z,1’Z)-(naphthalene-1,5-diylbis (Azaneylylidene)) bis (Methaneylylid ene))bis(N,N-dimethylaniline) (NSB)

4-(dimethylamino)benzaldehyde (DAB) (0.298 g, 2 mmol) and naphthalene-1,5-diamine (DAN) (0.214 g, 1 mmol) were dissolved in 50 mL ethanol with the addition of a few drops of glacial acetic acid followed by stirring for 12 h at 80 °C [25]. The forming yellowish solution was cooled to room temperature and the formed precipitate was filtered, and washed with cold diethyl ether, pale-yellow powder was collected with 90% yields of 4,4’-((1Z,1’Z)-(naphthalene-1,5-diylbis (azaneylylidene)) bis(methaneylylidene))bis(N,N-dimethylaniline) (Scheme 1), melting point: 315 °C. ^1^ H-NMR (D_2_O, 400 MHz, Fig. 1a): δ 8.4 (d, 1 H, CH = N), 8.19 (d, 1 H, naphthalene moiety), 7.44 (d, 1 H, naphthalene moiety), 7.07 (d, 1 H, naphthalene moiety), 7.92 (d, 1 H, benzene moiety), 6.71 (d, 1 H, benzene moiety moiety),3.08 (s, 3 H, −CH_3_). FTIR (KBr, Fig. 1b), υ 3027 cm^–1^ (= C–H), 2910 cm^–1^ (C–Has), 2805 cm^–1^ (C–Has), 1618 cm^–1^ (CH = N), 1527 cm^–1^ (C = C), 1200 cm^–1^ (C–N), and 819 cm^–1^ (para-ph).

#### Synthesis of 4,4’-((1Z,1’Z)-(naphthalene-1,5-diylbis(Azaneylylidene)) bis(Methaneylylid ene))bis(N,N-dimethyl-N-octylbenzenaminium)Dibromide (NCSB)

In a 250 mL round bottom flask that had been dried and filled with nitrogen, NSB (0.5 g, 1.1 mmol) in ethanol solution (50 mL) was added. Then octyl bromide (0.239 mL, 2.2 mmol) was taken up in a syringe and introduced and the solution slowly heats at 80 ^o^C for 48 h. After the reaction was completed, a pale-green precipitate was filtered and washed with ethanol before being recrystallized from diethyl ether to obtain a greenish powder with a 75% yield of 4,4’-((1Z,1’Z)-(naphthalene-1,5-diylbis(azaneylylidene))bis(methaneylylidene))bis(N,N-dimethyl-N-octylbenzenaminium)dibromide with melting point of 312.5 °C [26]. ^1^ H-NMR (D_2_O, 400 MHz, Fig. 1a): δ 8.4 (d, 1 H, CH = N), 8.19 (d, 1 H, naphthalene moiety), 7.44 (d, 1 H, naphthalene moiety), 7.09 (d, 1 H, naphthalene moiety), 7.91 (d, 1 H, benzene moiety), 6.8 (d, 1 H, benzene moiety moiety), 3.04 (s, 3 H, −CH_3_), 2.57 (t, 2 H, −N^+^CH_2_ CH_2_), 1.66–216 (m, 2 H, −CH_2_(CH_2_)_6_CH_3_), 1.24 (t, 3 H, −CH_2_(CH_2_)_6_CH_3_). FTIR (KBr, Fig. 1b), υ 3030 cm^–1^ (= C–H), 2910 cm^–1^ (C–Has), 2805 cm^–1^ (C–Has), 1608 cm^–1^ (CH = N), 1594 cm^–1^ (C = C), 1200 cm^–1^ (C–N), and 805 cm^–1^ (para-ph). MS: m/z calcd for C44H62Br2N4 [M + H]^+^: 808.3302, found: 808.7300 (Fig. 1c).

### Critical Micelle Concentration (CMC)

To study the surfactant properties of the synthesized naphthalene cationic Schiff base (NCSB), the CMC was determined via electrical conductivity measurements at different concentrations using a Cond-3210 SET Tetra-Con 325 method at 25° ± 1 °C. The CMCs were determined by plotting the specific conductivity against surfactant concentration at the intersection point. The standard free energy of micellization ($\varDelta {G}_{mic}^{o}$) per mole of CMC was calculated using the Eq. (1) [27]:

1 $$\Delta G_{mic}^o = \left( {2 - \beta } \right)RT\,{\rm{ln}}\,{C_{CMC}}$$

Where *β, R* and *T* are the degrees of counter ion dissociation, gas constant, and temperature, respectively, and C_*CMC*_ is the surfactant molarity.

### Procedures for Mn^2+^ and Co^2+^ Detection

Stock solution of NCSB sensor (10 µM) and 1000 µM of Fe^2+^, Cu^2+^, Mg^2+^, Co^2+^, Ni^2+^, Zn^2+^, Cd^2+^, Hg^2+^, Pb^2+^, Sn^2+^, Cr^3+^ and Mn^2+^ metal ions were prepared in distilled water. To explore the sensing performance of NCSB towards Mn^2+^ and Co^2+^, 1500 µL of the NCSB (10 µM) in distilled H_2_O was mixed with 1500 µL of various concentrations of Mn^2+^ and Co^2+^ to the final volume of 3 mL. Before detection, the samples were incubated at 25 °C for 5 min and the emission intensity was measured at the wavelength ranging from 300 to 700 nm (λex = 350 nm). To investigate the selectivity of the NCSB sensor towards Mn^2+^ and Co^2+^, the fluorescence emission of NCSB in the presence of Fe^2+^, Cu^2+^, Mg^2+^, Co^2+^, Ni^2+^, Zn^2+^, Cd^2+^, Hg^2+^, Pb^2+^, Sn^2+^, Cr^3+^ and Mn^2+^ ions (1000 µM) [28].

### Fabrication of Paper Analytical Device Combined with Smartphone

Adobe Illustrator software was employed to design circular zones with a diameter of 0.5 cm and the designed patterns were printed on Whatman No.1 filter paper via a Xerox ColorQube 8570 wax printer. Then the printed paper was heated in an oven at 120 °C for 2 min to melt the printed wax through the paper and form a hydrophobic barrier. The NCSB sensor was applied to filter paper (10 µM) and the filter paper was then allowed to dry at room temperature to create the NCSB paper-based sensor. A 10 µL of different concentrations of Mn^2+^ and Co^2+^ (0.5 to 125 µM) were dropped on the testing zone of paper sensors to detect Mn^2+^ and Co^2+^ in aqueous solution [29]. The visualized fluorescence images under 365 nm UV light were captured using a smartphone and the PAD Analysis phone application was used to analyze the fluorescence spots in the images, and the average intensity of red, green, and blue color was measured from the RGB channels. Using NCSB-based paper devices, the R/G values were used to determine Mn^2+^ and Co^2+^ concentrations.

### Determination of Mn^2+^ and Co^2+^ in Environmental Water

Environmental water (River and tap water) was used to test the possible application of the proposed sensor for Mn^2+^ and Co^2+^ analysis in a real environment. River water samples were obtained from the Nile River (Cairo, Egypt) and filtered twice to eliminate any solid materials followed by centrifuging at 4000 rpm for 15 min. The spiked river and tap samples were diluted 50 times with various concentrations of Mn^2+^ and Co^2+^ (0.5, 5, 10, and 25 µM) before being added to 10 µM NCSB sensor solution. Before detection, the samples were incubated at room temperature for 5 min and the fluorescence spectra were recorded under the same conditions mentioned above [30].

### Theoretical Calculations

For theoretical calculations, the Gaussian 09 package was used. The geometries in the ground state were optimized using density functional theory (DFT) and single-point computations. Becke’s three-parameter hybrid functional B3LYP was used for all calculations, with a 6–31*G(d,p) basis set for the NCSB and its complexes with Mn^2+^, and Co^2+^ metal ions [31].

### Response Surface Methodology (RSM) Analysis

The RSM technique was used to design the experiments, which looked at the interactions between independent factors and the expected response based on fluorescence intensity measurements. The pH values and time (min.) were the independent parameters (input-variables), while the fluorescence intensity (a.u.) of the two cations (Mn^2+^ and Co^2+^) were the response variables (output-response). A mathematical model produces the dependent variable fluorescence intensity. The RSM model’s performance was statistically predicted using ANOVA and R^2^ values [32].

## Results and Discussions

### Characterization of the Synthesized NCSB

Schiff bases are chelating ligands with a high affinity for various heavy metal ions due to their binding groups of imine group (–C = N–); as a result, they are used as chromophores to detect multiple cations. The studied naphthalene cationic Schiff base (NCSB) was synthesized in two steps by combining naphthalene-1,5-diamine with 4-(dimethylamino)benzaldehyde (DAN), followed by quaternization with octyl bromide, as shown in Scheme 1. FTIR and ^1^ H-NMR analysis were used to describe the chemical structure of NCSB, as shown in the experimental section. In the ^1^ H-NMR spectrum of NCSB, a singlet peak for the imine group (C = N) was found at 8.4 ppm, however the signal for the aromatic protons of NCSB was recognized as widened multiples between 7.09 and 8.19 ppm, which are attributed to the naphthalene ring replacements. The aromatic ring replacements are responsible for the 12 aromatic protons that appear between 6.8 and 7.91 ppm, whereas the octyl groups with 17 hydrogen protons appear between 1.24 and 3.04 ppm. In FTIR spectrum the aromatic protons (= C-H) and imine (C = N) groups related to NCSB were detected as stretching vibration signals at around 3030 and 1608 cm^− 1^, respectively [33]. The stretching vibrational signals for the C-H and C = C groups of NCSB were also observed at roughly 2910 and 1594 cm^− 1^, respectively.

### Critical Micelle Concentration and Optical Properties Studies NCSB

**Fig. 2:**
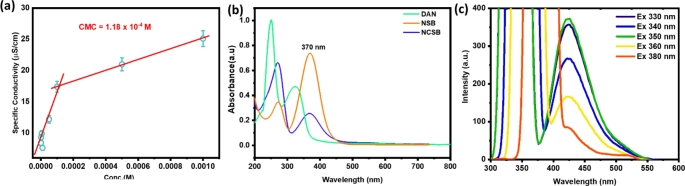
**(a)** The specific conductivity against surfactant concentrations plot for CMC determination. **(b)** Absorption spectra of DAN, NSB and NCSB (100 µM). **(c)** Fluorescence intensity of NCSB at different excitation wavelength in aqueous solution

The critical micelle concentration (CMC) of a surfactant in a bulk solution is the concentration at which micelles (surfactant molecule aggregation) form. The CMC is a significant attribute that may be calculated using conductivity measurements. Figure 2a shows the difference in conductivity for NCSB surfactant at varied concentrations. The break points in the specific conductivity (*K*) against surfactant concentration (*C*) graphs were used to calculate the CMC value for NCSB, which was 118 µM. The ratio of the two slopes also gave the degree of counterion dissociation value (*β*). The standard free energy of micellization ($\varDelta {G}_{mic}^{o}$) per mole of CMC was calculated using the Eq. (1). The acquired change in free energy for micellization ($\varDelta {G}_{mic}^{o}$) of the synthesized NCSB is negative (− 16.067 kJ mol^− 1^), showing that the micellization process is favorable and spontaneous in terms of reducing the resulting solution energy because of micelle dissolution in aqueous medium [34].

Figure 2b shows the UV-absorption spectra of 4-(dimethylamino)benzaldehyde (DAN) in chloroform solution, NSB in chloroform solution, and 50 µM of NCSB in H_2_O. DAN has a distinctive absorption band at 320 nm that redshifts to 370 nm after Schiff base production (NSB) due to an increase in the conjugated π-π* electronic transition. The maximum absorption band of the blue-colored NCSB lies at 370 nm, which can be attributed to intramolecular charge transfer transitions (ICT) between benzene ring of naphthalene moiety and imine (C = N) sites. ICT will be influenced by the substituted groups present in the benzene ring of DAB and DAN. In particular, the ICT transition in the absorption spectra showed a gradual redshift in the case of NSB and NCSB compared with DAN, and the absorption intensity of the NCSB band also decreased gradually. This indicates a strong electronic coupling between the para-substituted DAB and DAN, as the electron-donating ability increases in the ground state. Furthermore, the creation of cationic charges on the two terminal nitrogen atoms may induce a decrease in the strength of the NCSB absorption band at 350 nm and an increase in the intensity of the absorption band at 320 nm. The naphthalene cationic Schiff base probe (NCSB) fluorescence emission spectra were also studied and are shown in Fig. 2c. The strongest emission at 426 nm was discovered at a wavelength of 350 nm when NCSB was stimulated at several wavelengths between 330 and 380 nm [35].

### Selectivity of NCSB

**Fig. 3:**
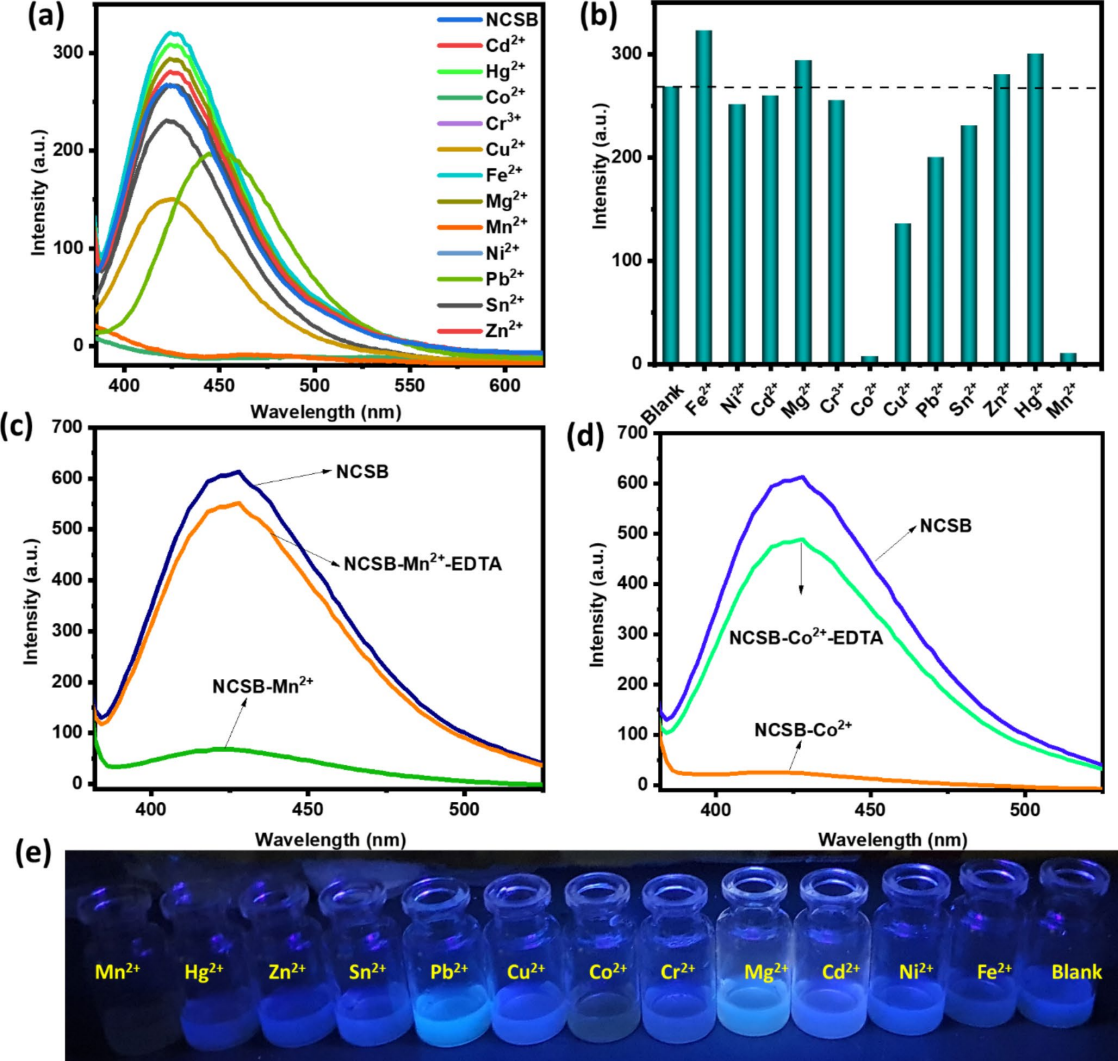
**(a, b)** Fluorescence spectra of NCSB in the absence and presence of various metal ion solutions (λ_ex_ = 350 nm). **(c, d)** Reversible changes in fluorescence intensity of NCSB (250 µM) after the sequential addition of Mn^2+^, Co^2+^ ions and EDTA in aqueous solution (λ_ex_ = 350 nm). **(e)** Photographs of aqueous suspensions (100 µM) of NCSB with and without addition of different metal ions solution under 365 nm UV light irradiation

To study the selectivity of NCSB sensor towards Mn^2+^ and Co^2+^ spectrofluorometric titration experiments were employed. The fluorescence properties of NCSB solutions with and without of 12 different metal ions including 1000 µM from each of Fe^2+^, Cu^2+^, Mg^2+^, Co^2+^, Ni^2+^, Zn^2+^, Cd^2+^, Hg^2+^, Pb^2+^, Sn^2+^, Cr^3+^ and Mn^2+^ are shown in Fig. 3a. Surprisingly, only Mn^2+^ and Co^2+^ significantly reduced the fluorescence emission of the NCSB sensor, whilst other cations had no effect on the fluorescence intensity of the NCSB sensor (Fig. 3b). Overall, 98.1 and 97.0% of the total fluorescence intensity of the NCSB was quenched upon the addition of Mn^2+^ and Co^2+^ ions, respectively. We think that the fluorescence quenching process is driven by significant complexation between the NCSB sensor and Mn^2+^ and Co^2+^ metal ions via the imine (–C = N–) and quaternary ammonium (R_4_N^+^) groups. To test whether the sensing performance of NCSB is reversible, 1 equiv. of a disodium salt of ethylenediaminetetraacetic acid (Na_2_EDTA) solution was added to the NCSB solution, which was incubated with 1 equiv. of a Mn^2+^ and Co^2+^ solution. After the addition of an Na_2_EDTA solution, the initial emission intensity of NCSB was almost recovered immediately from fluorescent NCSB-Mn^2+^ and NCSB-Co^2+^ (Fig. 3c,d). This experiment confirmed that the binding manner of NCSB with Mn^2+^ and Co^2+^ is reversible and the prepared sensor can be facilely regenerated. Moreover, the addition of Mn^2+^ and Co^2+^ resulted in a visible color shift from blue solution to colorless as given in Fig. 3e. Nonetheless, the addition of other metals to the NCSB sensor had no discernible effect on the color of the solution. NCSB clearly functions as a colorimetric chemosensory with high specificity for Mn^2+^ and Co^2+^ over other heavy metal cations [36].

### Optimization of the Sensing System

**Fig. 4:**
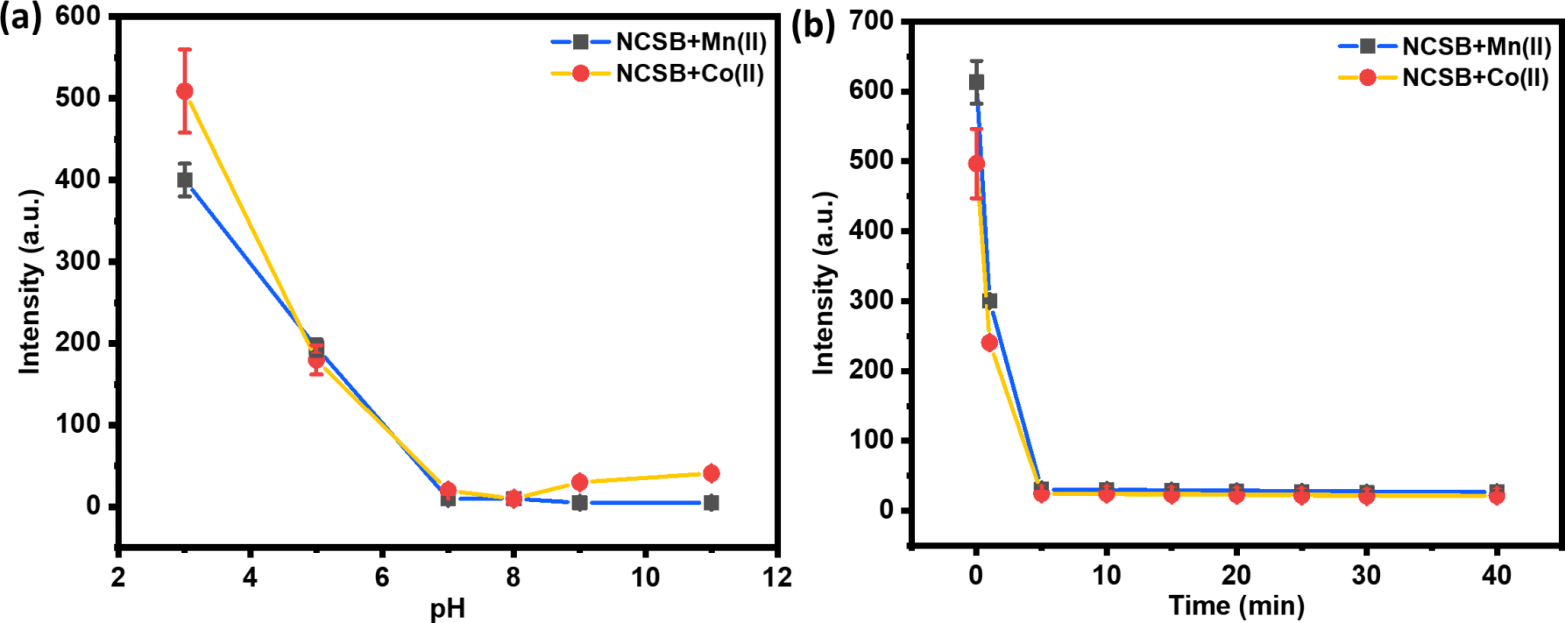
Optimization of the detection conditions. **(a)** Effects of the pH, **(b)** effects of the incubation time on the fluorescence intensity of NCSB (10 µM) in the presence of Mn^2+^ and Co^2+^ ions (100 µM), (λ_ex_ = 350 nm)

In order to enhance the NCSB sensor’s fluorescence characteristics towards the heavy metal ions, the pH and response time parameters were primarily investigated. In the presence of 100 µM of Mn^2+^ and Co^2+^ metal ions, the effect of pH solutions on the NCSB sensor’s intensity was investigated; the results are shown in Fig. 4a. In the pH range of 7 to 11, the complexation of the particular metal ions with the NCSB sensor led to the maximum quenching of fluorescence intensity. This result might be explained by the complexation of the metal ions Mn^2+^ and Co^2+^, and the fact that the proposed NCSB sensor was more stable at neutral and basic pHs than acidic ones. The imine group in NCSB is anticipated to be fully protonated at pH values below 5, which leads to mild complexation with metal ions [37]. Furthermore, to evaluate the influence of time on the heavy metal cations detection, 100 µM of Mn^2+^ and Co^2+^ solutions were incubated with 10 µM of NCSB solution, and fluorescence spectra were taken between zero and 40 min. As seen in Fig. 4b, the emission of the NCSB sensor gradually decreased with the injection of Mn^2+^ and Co^2+^. After 5 min of incubation, there was no discernible change in NCSB emission, hence 5 min was determined to be the appropriate duration for the complexation reaction between the NCSB sensor and the Mn^2+^ and Co^2+^ ions.

### Analytical Performance for Mn^2+^ and Co^2+^ Detection

**Fig. 5:**
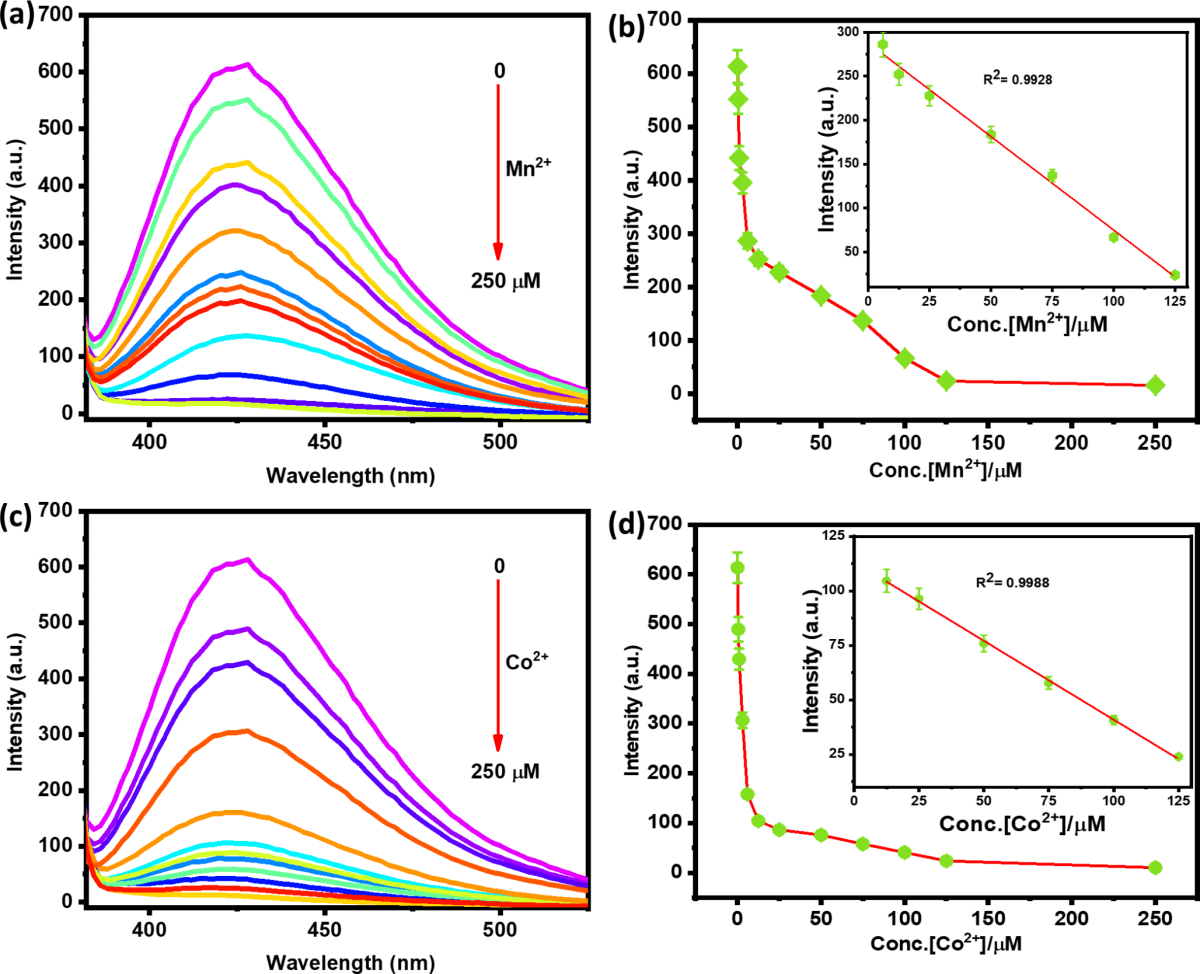
Fluorescent spectral changes of **NCSB** (10 µM) upon sequential addition of **(a, c)** Mn^2+^ Co^2+^ions (0–250 µM) in aqueous solution (λex = 350 nm, slit: 5). **(b, d)** The relationship between the fluorescence intensity of NCSB and the various concentration of Mn^2+^ and Co^2+^. Insets: corresponding linear regression analysis of intensity vs. [Mn^2+^] and [Co^2+^]

As shown in Fig. 5, the Mn^2+^ and Co^2+^ ions were detected in the aqueous solution under the aforementioned optimal pH and incubation conditions. As Mn^2+^ and Co^2+^ concentrations rose, the NCSB sensor’s intensity steadily dropped, as seen in Fig. 5a&c. There is a strong linear association between the emission intensity and the different Mn^2+^ and Co^2+^ concentrations in the ranges of 6 to 125 µM and 12.5 to 125 µM, respectively. The equations y = -2.130 x + 287.953 (R^2^ = 0.9928) and y = -0.7234 x + 113.280 (R^2^ = 0.9988) for Mn^2+^ and Co^2+^ detection, respectively, are plausible proof of this linearity (Fig. 5b&d). The limits of detection (LOD) based on 3σ/k was estimated to be as low as 0.014 µM (14.08 nM) for Mn^2+^ and 0.041µM (41.47 nM) for Co^2+^ [26, 38]. It is noteworthy that the LOD values are ~ 517 and 41 times lower than the permitted level of Mn^2+^ (7.28 µM) and Co^2+^ (1.7 µM) [6] in drinking water as defined by the World Health Organization (WHO) [39]. Hence, the NCSB has a far LOD than that required by the WHO guideline and could be a good tool for the detection of Mn^2+^ and Co^2+^ in environmental water. These findings demonstrate that the NCSB sensor possess a strong sensitivity and specificity for discriminating Mn^2+^ and Co^2+^ ions with a definite color change likely useful for detection with the naked eye.

### Comparison of the Current Method with Other Probes

Table 1 compares the sensitivity of our method to other published methods for Mn^2+^ and Co^2+^ detection. Although some methods are very sensitive, they have limitations such as low selectivity, extended analytical periods, the use of hazardous chemical solvents, or sensing that is not entirely in aqueous fluids [40–43]. The suggested NCSB sensor platform does not require any sophisticated procedures or expensive equipment, and it has high sensitivity, specificity, and a short detection time (less than 5 min). Furthermore, the structure of the developed Schiff base surfactant-based sensor was critical in achieving higher sensitivity than previously described approaches. The increased molecular interaction features generated by complexation with -C = N-, R_4_N^+^, and counter ions are commensurate with the suggested sensor’s sensitivity. These interactions are anticipated to increase sensor affinity for Mn^2+^ and Co^2+^ recognition, as well as quenching effectiveness.

### Quenching Mechanism Study

**Fig. 6:**
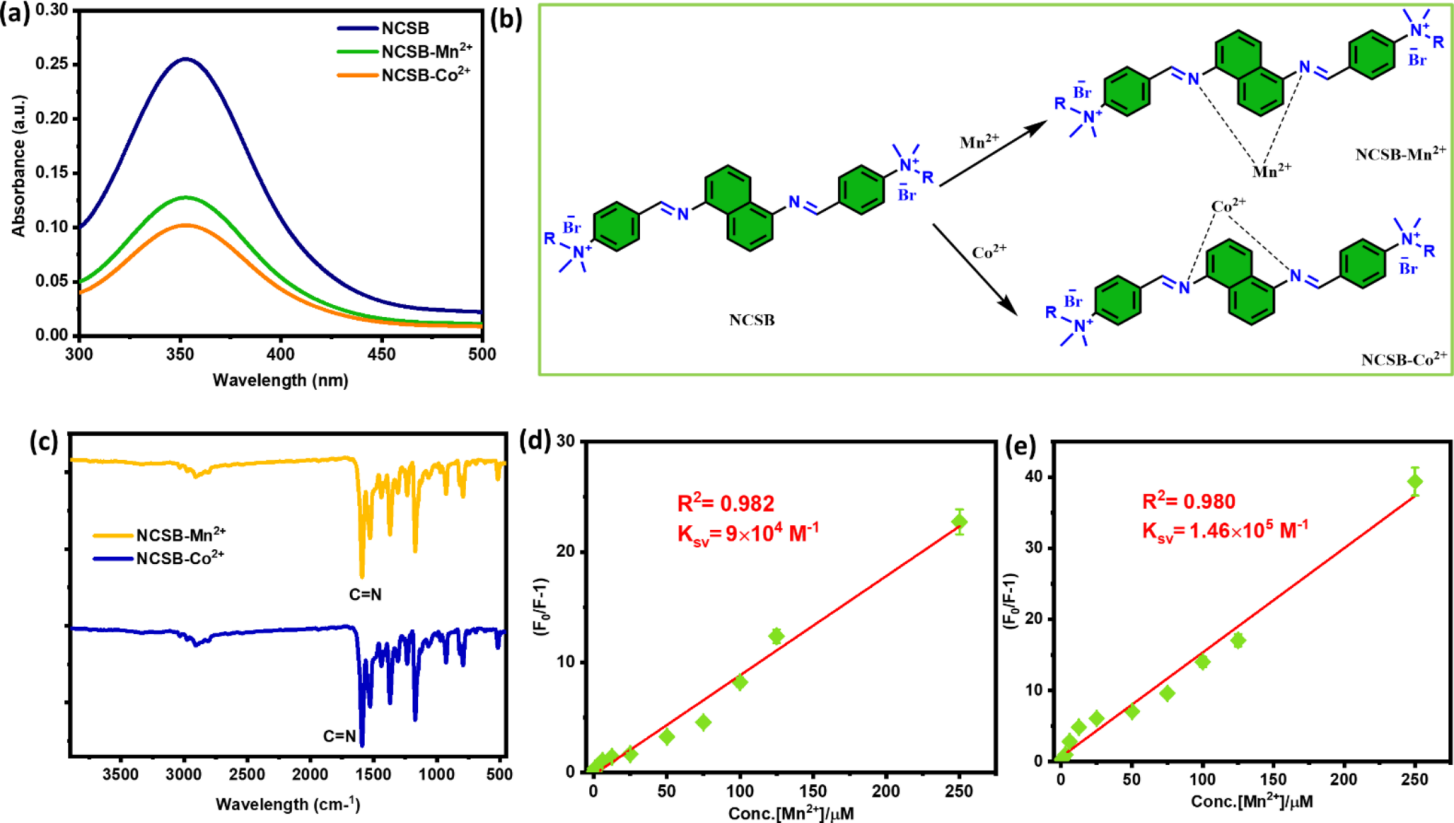
**(a)** Absorption spectra of NCSB and NCSB-Mn^2+^, and NCSB-Co^2+^, **(b)** Proposed Mn^2+^ and Co^2+^ sensing mechanism, **(c)** FTIR spectra of NCSB-Mn^2+^, and NCSB-Co^2+^ complexes, **(d, e)** Stern-Volmer plot for Mn^2+^ and Co^2+^ ions under studied NCSB sensor

The quenching mechanism can be classified as static, dynamic, or a combination of the two. The collisional quenching process only affects the fluorophores’ excited states. Static quenching, on the other hand, affects the ground state, resulting in the development of a ground-state complex between the NCSB fluorophore and the Mn^2+^ and Co^2+^ quencher, resulting in the formation of a nonfluorescent complex. Generally, static quenching is likely to occur by a shift in the absorption spectrum of the fluorophore [44]. As a result, the perturbation of the NCSB sensor’s absorption (see Fig. 6a) is recognizable in the presence of Mn^2+^ and Co^2+^ as a quencher, indicating the static quenching mechanism take occurred. Additionally, the absorption maxima near 370 nm caused by the π-π̽ and n-π̽ electronic transitions underwent slight redshifts with the addition of Mn^2+^ and Co^2+^ ions (see Fig. 6a). The active d-d transitions and charge transfers may be the cause of the observed color quenching in the NCSB-Mn^2+^ and NCSB-Co^2+^ complexes. Furthermore, due to photo-induced electron transfer, the ligand displays emission intensity near 426 nm at excitation wavelength 350 nm. The promotion of PET may be the cause of the significant quenching in NCSB emission intensity observed with the addition of Mn^2+^ and Co^2+^. Therefore, it may be more effective to explain sensing mechanisms by combining charge transfer and PET for the detection of Mn^2+^ and Co^2+^ ions [25, 45, 46]. This finding indicates that the fluorescence quenching process is caused by the occurrence of a strong complexation between Mn^2+^ and Co^2+^ metal ions and the NCSB sensor via the imine (C = N) group (see Fig. 6b). The proposed sensing mechanism was observed by the FTIR spectra NCSB-Mn^2+^ and NCSB-Co^2+^ complexes for a clear understanding of the binding sites. The FTIR studies revealed that the characteristic stretching frequency for the C = N group of the NCSB at 1608 cm^−1^ was red shifted to 1591 cm^−1^ in the presence of Mn^2+^ and Co^2+^ (see Fig. 6c). The frequency shifting to a lower wavenumber signifies a higher polarization of the C = N bond, which may be due to coordination to the Mn^2+^ and Co^2+^ ions.

The relevant fluorescence quenching levels were determined using the Stern-Volmer equation to assess the extent of quenching for NCSB sensor by the increasing concentration from each Mn^2+^ and Co^2+^ ions as follows [47]:

2 $$\frac{{{F_0}}}{F} = {K_{SV}}\left[ C \right] + 1$$

where *F*_0_ and *F* are the intensities with and without Mn^2+^ and Co^2+^, K_sv_ is the quenching constant (M^− 1^), and [*C*] is the concentration of the Mn^2+^ and Co^2+^, respectively. The plots of *F*_0_/*F* versus Mn^2+^ and Co^2+^ concentrations of NCSB in Fig. 6d and e promote both static and dynamic quenching happening simultaneously [6]. The NCSB exhibited a downward trend toward the x-axis, which was supposed to be a dynamic quenching mechanism [6]. Furthermore, the *K*_SV_ values for Mn^2+^ and Co^2+^ were derived from the slope of the linear Stern-Volmer plot to be 0.09 µM and 0.146 µM, respectively. The *K*_SV_ result represents the fluorophore’s binding affinity as well as its quenching potential. Higher K_SV_ values indicate high affinity event-induced fluorescence quenching [48]. In comparison to Mn^2+^ ions, NCSB offers improved quenching of Co^2+^ ions.

### Computational Study

**Fig. 7:**
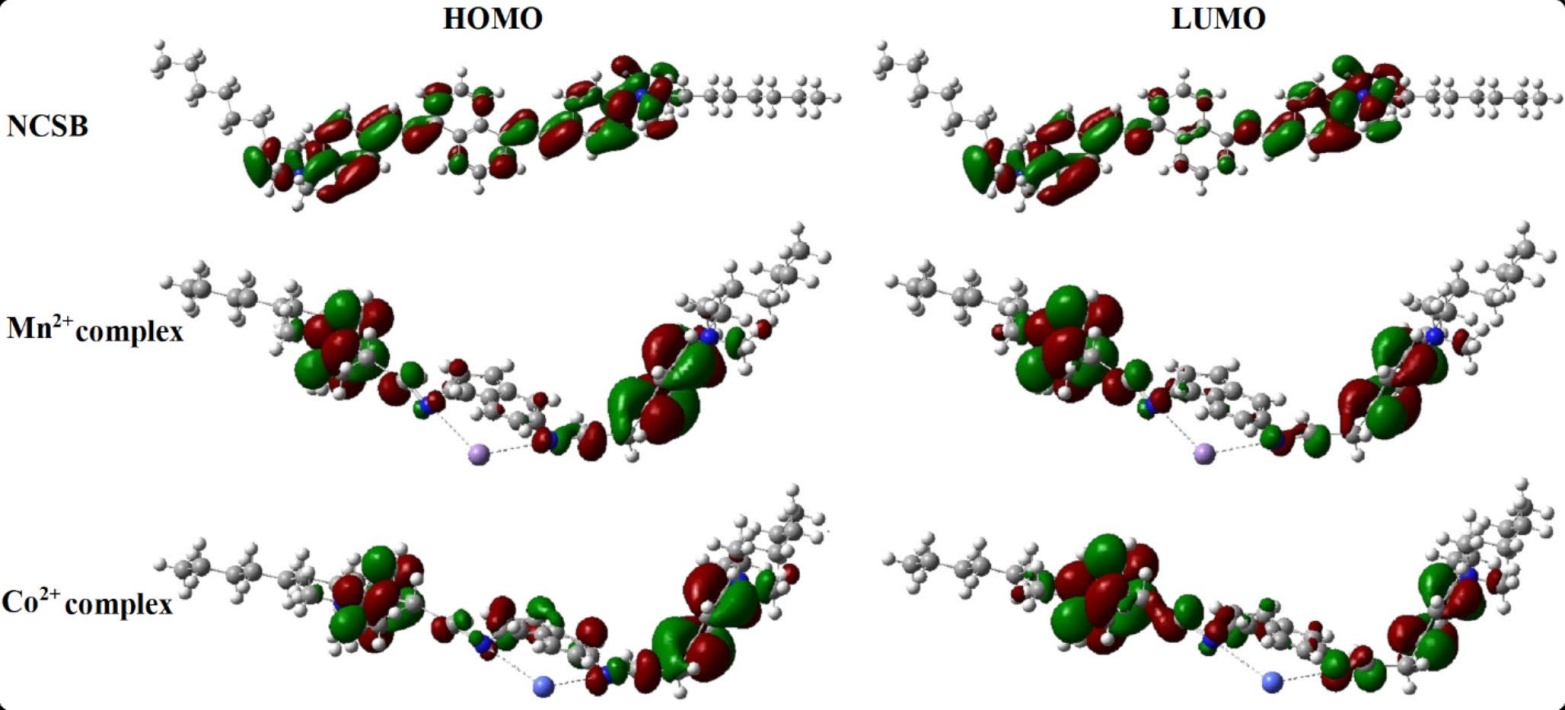
Electron distribution of highest occupied molecular orbital (HOMO) and lowest unoccupied molecular orbital (LUMO) energy levels of NCSB and its complexation with Mn^2+^ and Co^2+^ ions

Figure 7 depicts the electron distribution of NCSB sensor’s highest occupied molecular orbital (HOMO) and lowest unoccupied molecular orbital (LUMO) energy levels, as well as its complexation with Mn^2+^ and Co^2+^ ions. Table 2’s ordering of HOMO and LUMO energies, on the other hand, gives a qualitative estimate of electron excitation energies. A large energy gap shows that the NCSB is thermodynamically stable, but a small energy gap (Δ*E*) in the Mn^2+^ and Co^2+^ complexes enable an easy electronic transition, which is a favorable condition for fluorescence. The Mn^2+^ complex has a high possibility of electronic excitation at higher visible wavelengths due to its small energy gap. The electron distributions of the NCSB sensor’s HOMO and LUMO were − 1.3908 and − 1.1197, respectively. The HOMO side of the NCSB sensor has the greatest binding impact on metal ions. When the NCSB sensor was complexed with the target Mn^2+^ and Co^2+^, the Δ*E* values changed from 0.0010 eV for NCSB to 0.0080 eV for the Mn^2+^ complex and 0.0086 eV for the Co^2+^ complex. The NCSB sensor demonstrated strong electrostatic contact via functional groups like -C = N and -R_4_N^+^, which were responsible for complex formation with Mn^2+^ and Co^2+^ ions. The lower total energies of the complexed form Mn^2+^ and Co^2+^ than free NCSB demonstrated the robust stability of the complexed form of sensor. Thus, DFT calculations show that the complex with the lowest stabilization energy is more stable, and the stability is found to be in the order Mn^2+^ and Co^2+^ complex [49–55]. As a result, our experimental results from absorption and fluorescence tests are supported by these findings.

**Table 2:** Theoretical parameters of HOMO and LUMO Energy Gap for NCSB and its complexes

Parameters	NCSB	Mn^2+^ complex	Co^2+^ complex
*E*_HOMO_	-1.3908	-1.0691	-0.9777
*E*_LUMO_	-1.1197	-0.8506	-0.7429
Δ*E*	0.0100	0.0080	0.0086

### Potential Application as a Paper-Based Sensor

**Fig. 8:**
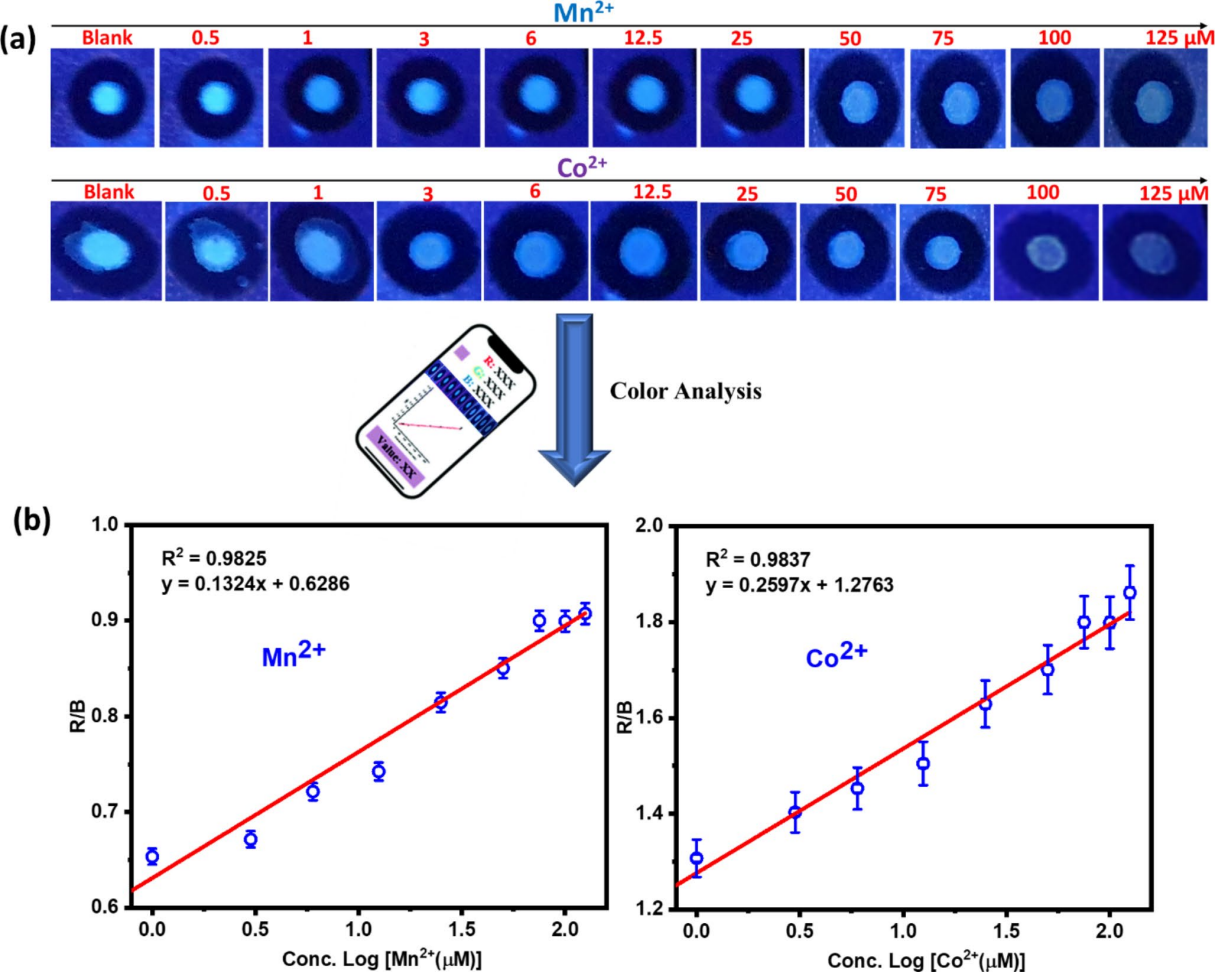
**(a)** Photographs of the paper-based devices (PAD) coated-NCSB sensor (100 µM, 10 µL) in the presence of different concentrations of Mn^2+^ and Co^2+^ ions (0-125 µM, 10 µL) under UV-light (365 nm). **(b)** Calibration plots using the PAD coupled with smartphone for the analysis of NCSB (100 µM) towards various concentrations of Mn^2+^ and Co^2+^ ions

We conducted additional paper analytical device (PAD) studies to prove the possibilities of applying the proposed strategy to analyze Mn^2+^ and Co^2+^ on-site. Mn^2+^ and Co^2+^ and NCSB sensor have been deposited on the filter paper detection zone. Under UV light, color changes on the detection zones can be seen with the naked eye for qualitative analysis. Subsequently, the RGB analysis was performed by capturing images with a smartphone and then scanning them with the “PAD Analysis” application to perform quantitative analysis [56, 57]. Figure 8a depicts images from the colorimetric assay of metal sensing using PAD device and the color changes can be easily noticed on the PAD to distinguish between Mn^2+^ and Co^2+^ metal ions. When the NCSB sensor and Mn^2+^ and Co^2+^ ions were coated in the same concentrations on the PAD detection zones, the color changed rapidly, resulting in significant fluorescence quenching. It is worth remarked that the variation of color appearance changed as the metal concentration increased from 0.5 to 125 µM. (Fig. 8a). In comparison to red (R) and blue (B) values, the color ratio of R and B intensity was chosen to establish the NCSB linear calibration plots with a good coefficient, r^2^ = 0.9825 and 0.9835 for Mn^2+^ and Co^2+^, respectively, (see Fig. 8b). The LOD for Mn^2+^ and Co^2+^ ions were calculated from PAD method to be 0.220 and 0.115 µM, respectively. As a result, the application of PAD as an efficient detection device in the detection of Mn^2+^ and Co^2+^ ions in the real samples were successfully demonstrated.

### Determination of Mn^2+^ and Co^2+^ in Environmental Water Samples

River and tap water samples were employed as complex matrices to further evaluate the potential application of the established NCSB sensor for Mn^2+^ and Co^2+^ detection in real samples. The river and tap water samples were first filtered to eliminate suspended matter before being investigated. In tap water samples, good recoveries of Mn^2+^ and Co^2+^ ranged from 95.95 to 105.91% with relative standard deviations (RSDs) ranging from 1.25 to 4.01%, were achieved as displayed in Table 3. Meanwhile, recoveries for spiked river water samples ranged from 94.82 to 100.2%, with RSDs ranging from 1.20 to 3.80%. These findings demonstrated the feasibility of the proposed method for accurately determining metal ions in environmental water samples, demonstrating its tremendous potential.

**Table 3:** Recovery test for Mn^2+^ and Co^2+^ detection environmental water samples using the proposed method

Analyte	Samples	Spiked, (µM)	Found, (µM)	Recovery, (%)	RSD, % (n = 3)
Mn^2+^	Tap water	0.5	0.48	95.95	2.55
		5	4.98	99.61	3.01
		10	10.59	105.91	4.01
		25	25.79	103.14	2.33
	River water	0.5	0.50	99.53	1.22
		5	4.96	99.28	2.02
		10	9.48	94.82	3.80
		25	24.38	97.50	3.22
Co^2+^	Tap water	0.5	0.50	100.2	1.25
		5	4.93	98.6	3.11
		10	10.01	100.1	1.98
		25	24.88	99.5	2.77
	River water	0.5	0.50	99.3	1.20
		5	5.01	100.2	3.55
		10	9.79	97.9	2.55
		25	24.35	97.4	2.41

### Surface Response Methodology (RSM) Optimization

The anticipated response fluorescence intensity of the R1 (for Mn^2+^) and R2 (for Co^2+^) were used for RSM analyses to design the experiment with 15 runs, as shown in Table 4. Using the RSM model, the experimental data were used to construct a quadratic model equation that relates the response fluorescence intensity to the input variables.

**Table 4:** Design matrix and RSM outcome of fluorescence intensity response for Mn^2+^ and Co^2+^ cations

Run	A	B	R1	R2
1	3	5	402.29	509.80
2	5	5	196.28	179.90
3	7	5	11.77	19.96
4	8	5	9.62	9.63
5	9	5	4.40	30.00
6	11	5	4.20	39.01
7	7	0	615.01	496.97
8	7	1	301.01	241.99
9	7	5	33.05	24.79
10	7	10	34.23	23.61
11	7	15	33.05	23.61
12	7	20	31.87	22.43
13	7	25	31.87	23.61
14	7	30	30.69	21.25
15	7	40	30.69	21.25

The equations below show the regression model equation in terms of the coded parameters:

3 $$Intensity{\rm{ }}\left( {M{n^{2 + }}} \right){\rm{ }} = {\rm{ }} - 496.92{\rm{ }} - 453.48{\rm{ }}A{\rm{ }} - {\rm{ }}93.74{\rm{ }}B{\rm{ }} + {\rm{ }}0.00{\rm{ }}AB{\rm{ }} + {\rm{ }}223.13{\rm{ }}{A^2} + {\rm{ }}249.95{\rm{ }}{B^2}$$

4 $$Intensity{\rm{ }}\left( {C{o^{2 + }}} \right){\rm{ }} = {\rm{ }}260.49{\rm{ }} + {\rm{ }}1662.30{\rm{ }}A{\rm{ }} - {\rm{ }}78.62{\rm{ }}B{\rm{ }} + {\rm{ }}0.00{\rm{ }}AB{\rm{ }} + {\rm{ }}1711.13{\rm{ }}{A^2} + {\rm{ }}203.60{\rm{ }}{B^2}$$

Intensity (Co^2+^) = 260.49 + 1662.30 A − 78.62 B + 0.00 AB + 1711.13 A^2^ + 203.60 B^2^ (4).

These equations can be used to make predictions about the reactions for specified amounts of each ingredient. The factors’ high levels are coded as + 1 by default, and their low levels as − 1. Both Eqs. 3 and 4 indicate the stability of the fluorescence intensity response with regard to the process variables of solution pH (A) and incubation time (B) within the studied range. The factor is coded as + 1 for high levels and 1 for low levels. This means that the combined effects have a positive sign, while the competing operation has a negative sign [58]. Table 5 displays the results of the ANOVA analysis of variance, which is also used to validate that the proposed model is appropriate for assessing the experimental data [59].

**Table 5:** Quadratic regression model ANOVA result of fluorescence intensity response for Mn^2+^ and Co^2+^ cations

Source	Sum of Squares		df		Mean Square		F-value		P-value	
	**Mn** ^**2+**^	**Co** ^**2+**^	**Mn** ^**2+**^	**Co** ^**2+**^	**Mn** ^**2+**^	**Co** ^**2+**^	**Mn** ^**2+**^	**Co** ^**2+**^	**Mn** ^**2+**^	**Co** ^**2+**^
Model	277,900	290,900	4	4	69,467	72,726	3.66	5.88	0.044	0.011
A	725	9736	1	1	725	9736	0.04	0.79	0.849	0.396
B	36,669	25,791	1	1	36,669	25,791	1.93	2.09	0.195	0.179
AB	0	0	0	0						
A²	387	22,765	1	1	387	22,765	0.02	1.84	0.889	0.205
B²	83,646	55,502	1	1	83,646	55,502	4.40	4.49	0.062	0.060
Residual	190,000	123,600	10	10	19,000	12,358				
Cor Total	467,900	414,500	14	14						
Predicted R²	-0.4952	-0.1167								
Adjusted R²	0.4315	0.5826								

**Fig. 9:**
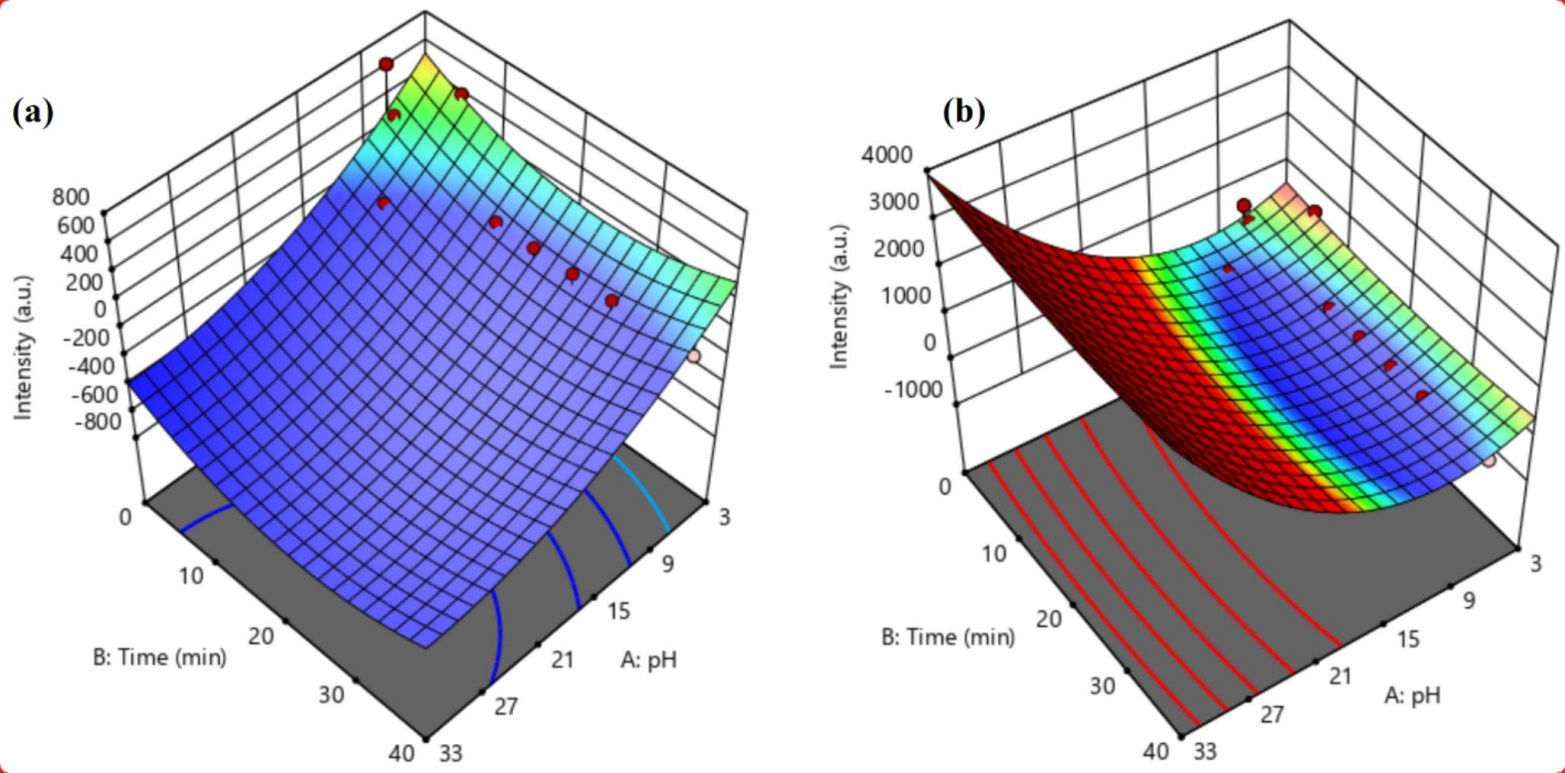
3D surface plots of outcome fluorescence intensity for: **(a)** Mn^2+^ cations, and **(b)** Co^2+^ cations

Significant coefficient factors have p-values less than 0.05, whereas insignificant coefficient factors have p-values greater than 0.05. The prediction in Table 5 suggests that the model terms A, B, AB, A2, and B2 are significant and were used. Furthermore, the ANOVA prediction of low p-values and high values of the model’s coefficient of determination (R^2^ = 0.5939 and 0.7018 for Mn^2+^ and Co^2+^ cations, respectively) confirmed the model’s importance and fitness. Although the fluorescence intensity response values decreased with the rising time, and lower, as shown in Fig. (9a,b). The complexation of the metal ions Mn^2+^ and Co^2+^ and the fact that the proposed NCSB sensor was more stable at neutral and basic pHs than acidic ones may both contribute to this result’s explanation. At pH levels below 5, the imine group in NCSB is projected to be entirely protonated, which results in a weak complexation with metal ions. Additionally, there was no noticeable change in NCSB emission after 5 min of incubation, hence 5 min was decided to be the ideal amount of time for the complexation reaction between the Mn^2+^ and Co^2+^ ions and the NCSB sensor. This established a strong relationship between this discovery and the experimental findings.

## Conclusion

A new naphthalene cationic Schiff base surfactant with two alkyl groups and two ammonium groups was successfully synthesized and characterized. Water solubility, strong optical properties derived from their conjugated backbones, and self-assembly behavior distinguish this type of cationic surfactant. The designed surfactant was utilized as a fluorescent sensor to determine the low level of the Mn^2+^ and Co^2+^ in water. The quenched fluorescence intensity with the fluorescent color change from blue to colorless under UV-lamp was observed after addition of Mn^2+^ and Co^2+^ ions therefore, it can be employed for naked eye detection. The fluorescence intensity of the NCSB quenches upon its interaction with Mn^2+^ and Co^2+^ ions, which was not seen by other ions thus demonstrating the high specificity of the NCSB. The NCSB sensor pushes down the LOD of the Mn^2+^ and Co^2+^ to be as low as 14.08 and 41 nM, respectively, which meet the detection limit approved by WHO. Interestingly, the NCSB sensor successfully demonstrated the ability for metal ion detection applying the paper sensor via color changes analysis on each detection zone. PAD method findings were also consistent with those achieved from fluorescent technique, indicating that PAD is appropriate for on-site metal ion analysis. Furthermore, the NCSB sensor can detect Mn^2+^ and Co^2+^ ion contaminants in river and tap water with satisfactory recoveries. Our finding of use a naphthalene cationic Schiff base surfactant in optical sensing opens a research path toward the fabrication of low-cost portable sensor with improved specificity and sensitivity. Our technique detects threats quickly (5 min) and does not require any expensive or complex procedures. Experimental interpretations are supported by theoretical calculations based on density functional theory (DFT) research. The linkages between the input elements and the anticipated response were evaluated using the quadratic model of the response surface methodology (RSM) modelling.

## Electronic Supplementary Material

Below is the link to the electronic supplementary material.

Supplementary Material 1

Supplementary Material 2

## Data Availability

The results/data/figures in this manuscript have not been published elsewhere, nor are they under consideration by another publisher. The data that support outcomes of this study are available on request from the corresponding author.
