# Supplementary material for: Fluorescence Naphthalene Cationic Schiff Base Reusable Paper as a Sensitive and Selective for Heavy Metals Cations Sensor: RSM, Optimization, and DFT Modelling
Source: J Fluoresc. 2023 Sep 15;34(5):2139–55. doi: 10.1007/s10895-023-03426-6 (PMC11445315; doi:10.1007/s10895-023-03426-6)
Supplement: Supplementary file 1 — Supplementary Material 1 [file 10895_2023_3426_MOESM1_ESM.docx]

**Highlights**

- A new naphthalene Schiff base surfactant (NCSB) was successfully synthesized.
- In aqueous solution, the NCSB Fluorescent sensor exhibits superior color change.
- The NCSB exhibited high sensitivity to Mn^2+^ and Co^2+^ cations.
- Paper-based sensor applied in environmental water with recoveries of 94.82-105.91%.
