# Supplementary figures and images for: Fluorescence Naphthalene Cationic Schiff Base Reusable Paper as a Sensitive and Selective for Heavy Metals Cations Sensor: RSM, Optimization, and DFT Modelling
Source: J Fluoresc. 2023 Sep 15;34(5):2139–55. doi: 10.1007/s10895-023-03426-6 (PMC11445315; doi:10.1007/s10895-023-03426-6)

**Graphical Abstract**


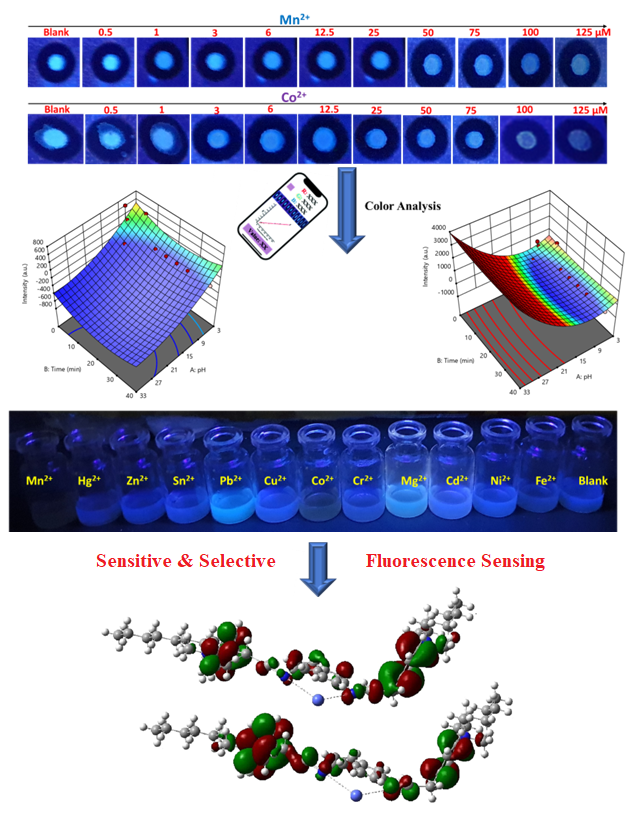

Supplement: Supplementary file 2 — Supplementary Material 2 [file 10895_2023_3426_MOESM2_ESM.docx]
